# Influence of Membrane Composition on the Passive Membrane Penetration of Industrially Relevant NSO-Heterocycles

**DOI:** 10.3390/ijms26157427

**Published:** 2025-08-01

**Authors:** Zsófia Borbála Rózsa, Tamás Horváth, Béla Viskolcz, Milán Szőri

**Affiliations:** 1 Institute of Chemistry, University of Miskolc, Egyetemváros A/2, H-3515 Miskolc, Hungary; tamas.horvath@uni-miskolc.hu (T.H.); bela.viskolcz@uni-miskolc.hu (B.V.); 2 Higher Education and Industrial Cooperation Centre, University of Miskolc, H-3515 Miskolc, Hungary

**Keywords:** passive membrane penetration, NSO heterocycles, lipid bilayers, molecular dynamics, AWH, membrane composition

## Abstract

This study investigates how phospholipid headgroups influence passive membrane penetration and structural impact of four nitrogen-, sulfur-, and oxygen-containing heterocycles (NSO-HETs)—N-methyl-2-pyrrolidone (PIR), 1,4-dioxane (DIOX), oxane (OXA), and phenol (PHE). Using all-atom molecular dynamics simulations combined with Accelerated Weight Histogram free energy calculations, the passive transport of NSO-HETs across DPPC, DPPE, DPPA, and DPPG bilayers was characterized. DPPG showed the highest membrane affinity, increasing permeability (logP_memb/bulk_) by 27–64% compared to DPPE, associated with the lowest permeability and tightest lipid packing. Free energy barriers are also decreased in DPPG relative to DPPE; PIR’s central barrier dropped from 19.2 kJ/mol (DPPE) to 16.6 kJ/mol (DPPG), while DIOX’s barrier decreased from 7.2 to 5.2 kJ/mol. OXA exhibited the lowest central barriers (1.2–2.2 kJ/mol) and uniquely accumulated at higher concentrations in the bilayer center than in bulk water, with free energy ranging from −3.4 to −5.9 kJ/mol. PHE and OXA caused significant bilayer thinning (up to 11%) and reduced lipid tail order, especially in DPPE and DPPA. Concentration effects were most pronounced in DPPE, where high solute loading disrupted lipid order and altered free energy profiles. These results highlight the crucial role of headgroup identity in modulating NSO-HET membrane permeability and structural changes.

## 1. Introduction

Biological membranes are crucial structures that encapsulate cells and enable a wide range of cellular functions. The core of these membranes is the lipid bilayer, composed of diverse lipid molecules that vary in size, chemical structure, and polarity. These lipids are amphipathic, containing a hydrophilic head group and lipophilic tail regions, both of which can differ in type, length, and degree of saturation ensuring the functional diversity of membranes [1,2,3]. Lipid headgroups can be both neutral—such as choline or ethanolamine—and anionic—such as serine, inositol, or glycerol, whose composition defines the surface charge of the membrane. The resulting electrostatic landscape influences not only permeability and protein function, but also the broader regulatory and communicative capacity of cellular membranes [4,5].

Eukaryotic membranes predominantly consist of glycerol-based lipids (GLs), sphingolipids (SLs), and sterols. Among the glycerol-based lipids, phosphatidylcholine (PC) and phosphatidylethanolamine (PE) are the most abundant, with smaller amounts of phosphatidylserine (PS), phosphatidylglycerol (PG), phosphatidic acid (PA), and cardiolipin (CL) contributing to the structural diversity of the membrane.

PC and PE are two of the most important neutral phospholipids found in all living organisms [2,3,4,6,7]. PC contains a choline headgroup, and PE contains a primary amine; both are zwitterionic under physiological conditions but differ in size and hydrogen bonding capacity [8,9]. PC lipids are the most abundant phospholipids of mammalian cell types and organelles, comprising about 40–50% of the total cellular phospholipids. PE is typically the second most abundant headgroup, especially in mitochondrial inner membranes, but it is also abundant in bacterial membranes [4,10,11]. Due to their small headgroup, PE molecules form extensive hydrogen bonding networks. This increases the phase transition temperature and stabilizes the membrane by reducing permeability and promoting tighter lipid packing. In mixed PC/PE membranes, increasing PE content further reinforces this ordering effect, leading to a more tightly packed and less fluid bilayer [6,9,12,13,14].

PG, in contrast, is an anionic lipid due to its glycerol–phosphate headgroup, making it a key contributor to membrane charge and interactions with cationic species. Its glycerol moiety also enhances hydrogen bonding and solvation, suggesting specialized structural roles beyond charge alone. It plays essential structural and functional roles in photosynthetic membranes of plants and cyanobacteria, and serves as a precursor for cardiolipin synthesis, which is critical for mitochondrial membrane integrity and activity in both plants and animals [5,7,15]. PA is a simple anionic phospholipid key to signaling, lipid metabolism, and membrane shaping [16].

While most compounds are theoretically capable of crossing lipid bilayers, the rate and likelihood of penetration depend heavily on molecular properties and the membrane environment [17,18]. Variations in lipid headgroup identity and proportion can significantly affect membrane penetration. This effect stems from differences in hydrogen bonding between lipid headgroups, which influence membrane packing, water exclusion, and, ultimately, solute permeability [19,20,21]. For example, liposomes containing POPC (1-palmitoyl-2-oleoyl-glycero-3-phosphocholine) mixed with POPA (1-palmitoyl-2-oleoyl-sn-glycero-3-phosphate) or POPE (1-palmitoyl-2-oleoyl-sn-glycero-3-phosphoethanolamine) exhibit reduced permeation of small ions compared to pure POPC, likely due to increased intermolecular hydrogen bonding between headgroups. Conversely, PG headgroups—with multiple hydroxyl groups—can increase water retention near the membrane surface, decreasing lipid packing and increasing leakage [22]. Similarly, studies of membranes with different ratios of DOPC (1,2-dioleoyl-sn-glycero-3-phosphocholine) and DOPE (1,2-dioleoyl-sn-glycero-3-phosphoethanolamine) show altered permeability profiles, with smaller molecules permeating less and larger molecules more [1]. Additionally, inclusion of POPS (1-palmitoyl-2-oleoyl-sn-glycero-3-phospho-L-serine) lipids in POPC bilayers raises the energetic barrier for neutral compounds [23]. These findings may underscore the crucial role of lipid headgroups in regulating passive membrane penetration.

Due to the complexity of biological membranes, molecular dynamics (MD) simulations have become an essential tool in membrane biophysics, providing atomic-scale insights into lipid organization, membrane deformation, and interactions with permeating compounds [24,25]. Several computational approaches have been developed to calculate the energetics of membrane penetration, such as Umbrella Sampling [26], Adaptive Biasing Force [27,28], Free Energy Perturbation [29], Metadynamics [30], and Accelerated Weight Histogram (AWH) [31,32,33,34].

AWH is a relatively recent method successfully used to determine the potential of mean force (PMF) for passive membrane penetration of small molecules [18,21,35]. AWH is an extended ensemble technique that applies an adaptive bias to flatten the free energy landscape, enabling efficient sampling of both low- and high-energy configurations. Importantly, the target distribution can be customized to focus sampling on regions of interest. Like other adaptive biasing approaches, AWH introduces a history-dependent potential to iteratively reduce free energy barriers along the reaction coordinate. Its main advantages include fast convergence—exponential in the initial phase—and a minimal requirement for fine-tuning. It performs reliably with only a few input parameters and shows low sensitivity to their exact values, making it a practical and efficient choice for membrane-related free energy calculations.

This study aims to systematically investigate how phospholipid headgroups influence the passive membrane penetration of industrially relevant cyclic compounds, and how the presence of these permeants affects membrane structure.

The family of cyclic hydrocarbons is diverse and includes nitrogen-, sulfur-, or oxygen-containing heterocyclic hydrocarbons (NSO-HETs) [36]. Since NSO-HETs contain lipophilic groups, the primary site of their toxicity is the cell membrane [37,38,39,40,41]. This membrane accumulation can lead to structural disruptions, also affecting membrane integrity [42,43] and potentially enhancing the permeability to other compounds [44,45,46]. Moreover, several NSO-HETs have been implicated in embryotoxicity and carcinogenicity, suggesting they must traverse the membrane to exert intracellular effects [36,47,48].

The selected four compounds—N-methyl-2-pyrrolidone (PIR), 1,4-dioxane (DIOX), oxane (OXA, also known as tetrahydropyran), and phenol (PHE)—are industrial solvents widely used across various sectors, often resulting in their presence as environmental contaminants. PIR is a polar, nitrogen-containing five-membered heterocycle extensively used in pharmaceuticals, paints, and adhesives, and also in the manufacture of lithium batteries [49,50,51]; DIOX is a six-membered oxygen heterocycle, widely used in the cosmetic industries, and also known for its persistence and carcinogenicity in water supplies [52,53]; OXA is a six-membered cyclic ether notable for its biodegradability which can be derived from renewable biomass, making it a greener alternative solvent and also tested as a biofuel additive [54,55]; and PHE is an aromatic compound with a hydroxyl group, prevalent as a toxic pollutant from petrochemical and industrial wastewater [56,57]. Toxicologically, PHE and DIOX are established carcinogens, while PIR is recognized for its reproductive toxicity; in contrast, OXA is considered non-toxic and biodegradable.

To isolate the effects of headgroup chemistry on the membrane penetration of the selected NSO-HETs, model membranes composed of four different, individual phospholipids were used: DPPC (1,2-Dipalmitoyl-sn-glycero-3-phosphatidylcholine), DPPE (1,2-Dipalmitoyl-sn-glycero-3-phosphoethanolamine), DPPA (1,2-Dipalmitoyl-sn-glycero-3-phosphate (phosphatidic acid)), and DPPG (1,2-Dipalmitoyl-sn-glycero-3-phosphoglycerol). All lipids share identical saturated dipalmitoyl (C16:0) acyl chains, ensuring that differences in permeability and membrane behavior arise solely from variations in headgroup structure. DPPC and DPPE are zwitterionic at physiological pH, whereas DPPA and DPPG carry a net negative charge. Additionally, DPPA (337 K) [58,59] and DPPE (339 K) [9,60] exhibit higher main phase transition temperatures than DPPC and DPPG (314 K), reflecting how headgroup interactions influence bilayer packing and thermal behavior.

**Figure 1:**
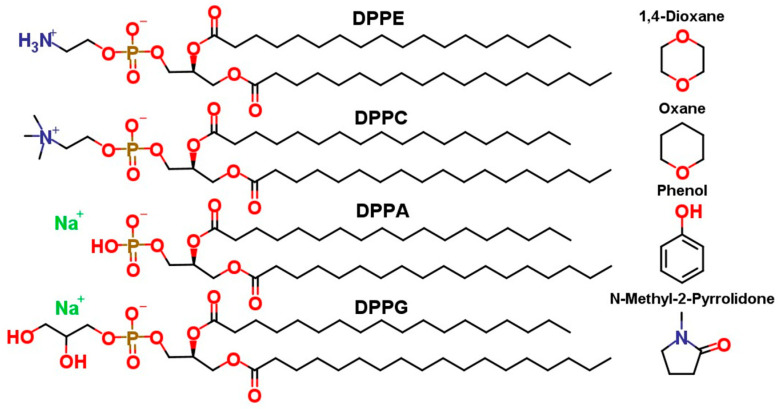
Two-dimensional structure of the investigated phospholipids (DPPE, DPPC, DPPA, and DPPG) and NSO-HETs such as N-methyl-2-pyrrolidone (PIR), 1,4-dioxane (DIOX), oxane (OXA), and phenol (PHE).

The molecules used in this study are shown in Figure 1. Passive membrane translocation was characterized by MD simulations in combination with the AWH method, for the calculation of PMF profiles along the membrane normal at two different concentrations. In parallel, classical MD simulations were used to assess membrane structural changes in response to the presence of permeants.

## 2. Results and Discussion

### 2.1. Membrane Structural Parameters

**Figure 2:**
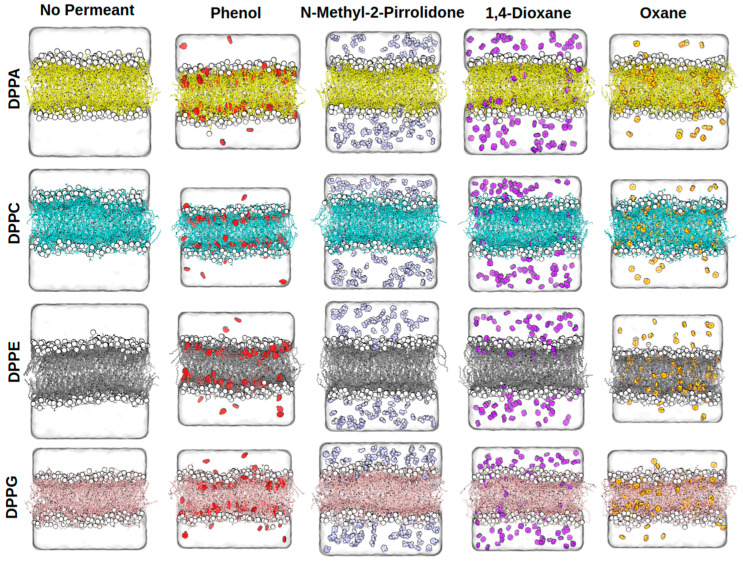
Snapshots of the equilibrium structures of the different membrane systems. In the figure, the colors represent the following membranes: yellow—DPPA, cyan—DPPC, grey—DPPE, pink—DPPG; and the following additives: red—PHE, ice blue—PIR, purple—DIOX, orange—OXA. Results were obtained by molecular dynamics simulations performed in this study using GROMACS 2023.2 and the CHARMM force field.

**Figure 3:**
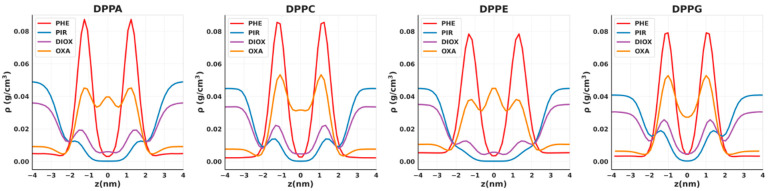
Mass density profiles of the solutes along the membrane normal in the investigated membrane models. Results were obtained by molecular dynamics simulations performed in this study using GROMACS 2023.2 and the CHARMM force field.

Snapshots of the equilibrated membrane systems are shown in Figure 2. All of the NSO-HET compounds investigated remain fully solvated in the aqueous phase without evidence of aggregation, consistent with their hydrophilic or amphiphilic character. Each solute permeates the lipid bilayers to varying extents, as quantified by the mass density profiles along the membrane normal, shown in Figure 3, and in all the remaining cases, the mass density profiles are shown in Supplementary Materials, Figure S2.

Among the compounds studied, PIR exhibits the least membrane insertion, localizing only as deep as the inner region of the headgroup zone, with negligible presence at the bilayer center. A small amount of DIOX penetrates the membrane, accumulating primarily at the inner site of the headgroup region, with a minor population reaching the bilayer center. Despite containing two symmetrically positioned, slightly polar ether oxygen atoms, DIOX has no permanent electric dipole moment and is considered overall nonpolar. Its limited insertion can be explained by the weak polar interaction sites. In contrast, OXA shows consistent accumulation both near the bilayer center and at the inner headgroup region, due to its increased lipophilicity compared to dioxane: the lipophilic ring supports deeper insertion, while the ether oxygen allows partial interaction near the interface. Phenol, on the other hand, is found exclusively at the inner site of the headgroup region. Its exposed hydroxyl group forms hydrogen bonds with lipid headgroups or interfacial water, but disfavors deeper penetration into the hydrophobic core. Despite the insertion of NSO-HET compounds, the overall membrane structures remain largely intact throughout the simulations. Only minor membrane undulations are observed, with no notable increase in amplitude or frequency in the presence of pollutants compared to pollutant-free bilayers. This suggests that the membrane integrity and liquid–crystalline phase are well preserved under all conditions studied.

While the overall behavior of the individual NSO-HETs is similar across the different phospholipid bilayers, subtle differences emerge depending on membrane composition. In DPPA and DPPE membranes, DIOX and OXA show distinct density peaks at the bilayer center, indicating unique accumulation patterns not observed in DPPC or DPPG. Additionally, pollutant density at the inner headgroup region is generally reduced in DPPE compared to other membranes. This reduction is especially pronounced for PIR, which shows a noticeably lower concentration inside DPPE compared to DPPC, DPPG, or DPPA membranes, as visible from the lack of a concentration peak at the inner site of the headgroups. Phenol’s spatial distribution remains consistent across all bilayers, although the magnitude of its density peaks near the headgroups is noticeably smaller in DPPE and DPPG. Furthermore, PIR and DIOX achieve their highest concentrations within the bilayer in DPPG and DPPC, where their density profiles display the most prominent peaks at the site of the headgroups, showing similar patterns as PHE and MOR. These observations suggest that phospholipid composition subtly modulates both the depth and localization preferences of these compounds within the membrane.

**Figure 4:**
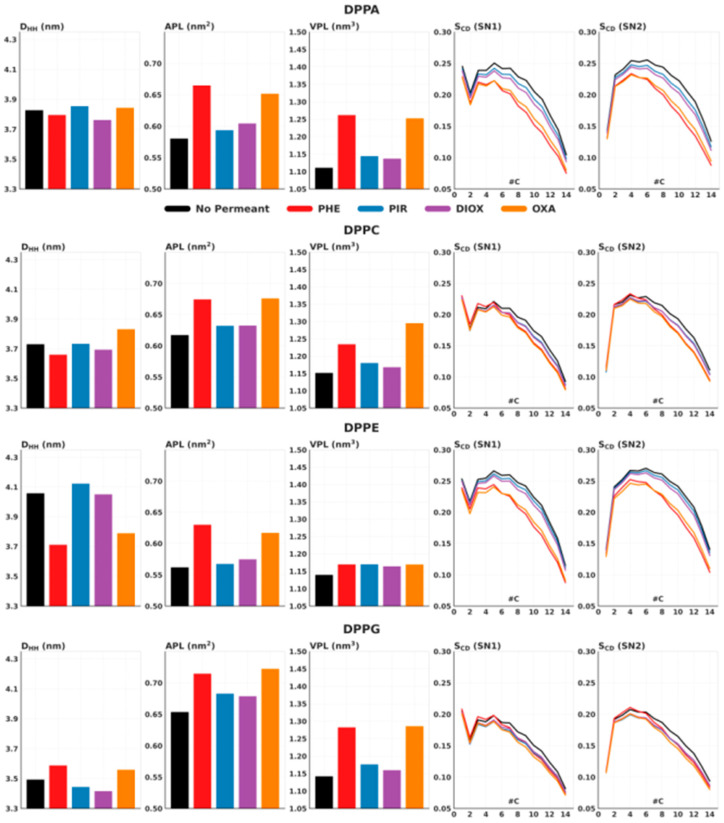
Membrane structural properties (D_HH_, APL, VPL, and S_CD_) of DPPC membranes in the presence of the investigated NSO-HET molecules—phenol (PHE), N-methyl-2-pirrolidone (PIR) 1,4-dioxane (DIOX) and oxane (OXA). Results were obtained by molecular dynamics simulations performed in this study using GROMACS 2023.2 and the CHARMM force field.

DPPA and DPPE membranes exhibit similar structural properties, characterized by relatively high bilayer thickness and tightly packed, highly ordered lipid tails, reflected in their small APL values (Figure 4). This similarity is partly due to their relatively small headgroup sizes, which promote more ordered and compact bilayers, as it was shown before [6,61]. In contrast, DPPC and DPPG contain larger headgroups, resulting in looser packing, less ordered lipid tails [12]. Among these, DPPG has the least ordered lipid tails, the smallest bilayer thickness, and the largest APL, while DPPC displays intermediate tail order and thickness values.

The presence of NSO-HET compounds leads to changes in membrane properties that vary depending on the compound and membrane composition (Figure 4). PHE and OXA cause the largest disturbances in bilayer structure due to their high concentrations within the membranes. They generally increase the APL value and decrease bilayer thickness as they accumulate at the inner site of the headgroups. These effects are particularly pronounced in DPPA and DPPE membranes, which initially exhibit greater bilayer thickness and more ordered lipid tails. In these membranes, PHE and OXA also significantly reduce the lipid tail order parameter (S_CD_), leading to less ordered tails, and more defined structural changes. In other membrane–pollutant combinations, the S_CD_ remains largely unchanged.

In contrast, DIOX and PIR penetrate the bilayers to a lesser extent and accumulate primarily at the inner site of the headgroups, showing minimal presence within the bilayer interior. Due to their lower bilayer concentrations, these compounds induce smaller structural changes, predominantly modest increases in APL. As the concentration of each pollutant within the bilayers is relatively consistent across membrane types, the observed structural effects depend primarily on compound-specific interactions rather than on the bilayer composition.

By using the obtained D_HH_ and the density distribution of the NSO-HETs, the membrane-bulk partition coefficient (P_memb/bulk_) can be calculated with the following equation [62]:

$$P_{memb/bulk}=\frac{\int_{{-D}_{HH}/2}^{{+D}_{HH}/2}\rho_{i}\left(z\right)dz/D_{HH}}{\left(\int_{-Z/2}^{+Z/2}\rho_{i}\left(z\right)dz-\int_{{-D}_{HH}/2}^{{+D}_{HH}/2}\rho_{i}\left(z\right)dz)/(Z_{box}-D_{HH}\right)}$$

where ρ_i_ is the density of the investigated additive and Z_box_ is the simulation box length along the membrane surface normal. LogP_memb/bulk_ values are shown in Table 1.

The octanol–water partition coefficient (logK_o/w_) is often used to estimate a compound’s membrane affinity. For the molecules studied here, logK_o/w_ values range from 1.48 [63] for PHE, 0.95 [64] for OXA, to –0.26 [65] and –0.38 [66] for DIOX and PIR, respectively. Experimental membrane/bulk partition coefficients are available for PHE in several systems: erythrocyte membranes (0.78) [63], plant cuticular membranes (1.51–1.59) [67], and SOPC bilayers (1.97) [68]. For phenol, the calculated logP_memb/bulk_ values (0.72–0.99) are in good agreement with this experimental range, supporting the reliability of the simulations.

The calculated logP_memb/bulk_ values (Table 1) also follow the overall logK_o/w_ trend. The results show that PHE and OXA prefer to partition into the membrane phase, while DIOX and PIR have negative logP_memb/bulk_ values, showing a preference for the aqueous phase. Among the NSO-HETs studied, PHE shows the strongest membrane affinity across all phospholipid types, with the highest value observed in the case of DPPG and the lowest for DPPE. On the contrary, PIR has the lowest membrane affinity, though it follows a similar trend to PHE with the lowest value in DPPG and highest in DPPE. DIOX shows the closest logP_memb/bulk_ values to zero across all membrane types, indicating only weak membrane association and a more even distribution between the water and membrane phases. OXA shows consistent membrane affinity, with values that remain relatively stable across the different membrane types. To summarize, the logP_memb/bulk_ values obtained for different membrane types show a strong linear correlation.

### 2.2. PMF Profiles of the Studied Systems

**Figure 5:**
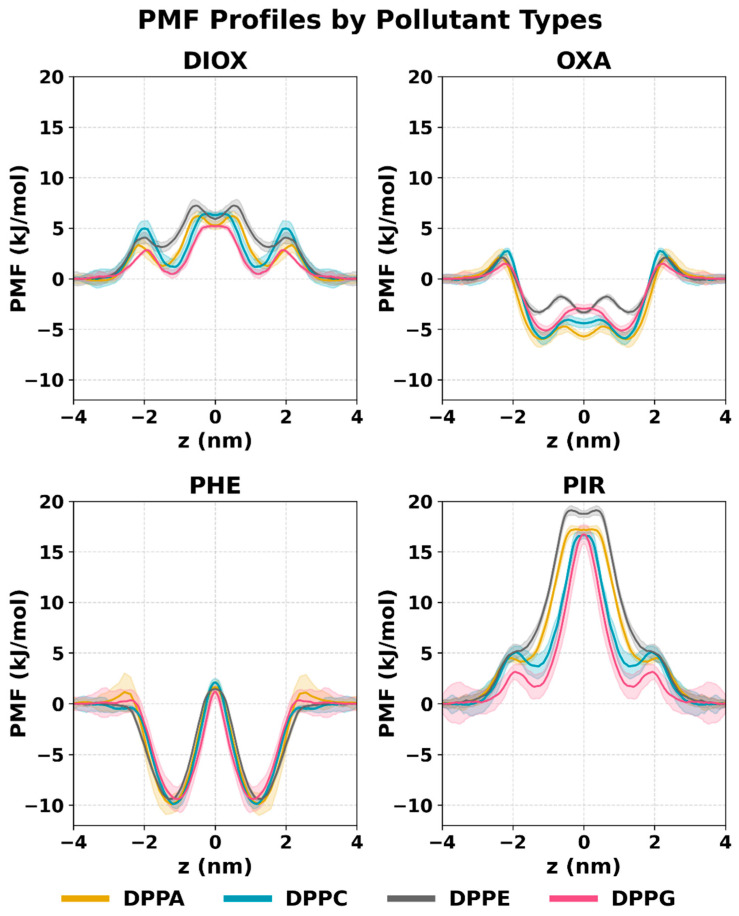
Free energy profiles of the compounds investigated in the presence of different phospholipids obtained by AWH simulations. Results were obtained by molecular dynamics simulations performed in this study using GROMACS 2023.2 and the CHARMM force field.

The membrane penetration free energy profiles of the investigated pollutants (Figure 5) are generally similar across all membrane types, with little variation due to membrane composition but clear differences between compounds. The membrane can be divided into four structurally distinct regions, based on the model introduced by Marrink and Berendsen [69]: (I) the perturbed interfacial water layer, where lipid–water interactions begin to influence solubility and diffusion; (II) the charged headgroup region, acting as the first energy barrier for polar compounds; (III) the dense acyl chain region, representing the main hydrophobic barrier to permeation; and (IV) the bilayer center, a low-density zone with increased free volume. These regions correspond well to typical features of the free energy profiles and offer a useful framework for understanding membrane penetration behavior.

Phenol exhibits two free energy minima located around 1.50–1.70 nm from the bilayer center, depending on the membrane type and thus the membrane thickness. These minima correspond to regions of higher phenol density near the inner site of the headgroups. The main energy barrier for phenol penetration is at the bilayer center, between 10.6 and 12.0 kJ/mol, consistent across all membranes. The profiles are very similar for all membrane types, with mostly overlapping standard deviations. The only exception is a small barrier (~1.1 kJ/mol) observed in DPPA membranes when penetrating the headgroups, likely due to the high charge density in that region.

PIR exhibits two main energy barriers in its PMF profiles: one at the site of the headgroups (~2.0 nm from the bilayer center) and a central barrier at the bilayer core. The outer barrier is relatively small, ranging from 3.2 to 5.1 kJ/mol, depending on the membrane type, with the lowest value observed in DPPG and the highest in DPPC and DPPE. The central barrier for PIR is the highest among all investigated compounds and membranes, reaching 19.2 kJ/mol in DPPE and 16.6 kJ/mol in DPPC and DPPG. Its width reflects the membrane thickness, being widest in DPPE and narrowest in DPPG. A metastable state is observed at the inner site of the headgroups (1.9–1.7 nm from the bilayer center) in all membrane types except DPPE, aligning with PIR’s mass density profiles. The absence of this energy minimum in DPPE is consistent with the lack of PIR accumulation inside these membranes.

The PMF profile of DIOX closely resembles that of PIR, featuring two main energy barriers at the headgroup region and at the bilayer center, separated by a metastable state at the inner site of the headgroups. The outer barrier, located around 2.0 nm from the bilayer center, ranges from 2.8 to 5.0 kJ/mol, with the highest value in DPPC and the lowest in DPPG. The central barrier is notably lower than that of PIR, between 5.2 and 7.2 kJ/mol, and follows a similar membrane-dependent trend: lowest in DPPG and highest in DPPE.

OXA displays the most complex PMF profile among the investigated compounds. A small outer barrier appears at the headgroup region, located between 2.0 and 2.3 nm from the bilayer center, with a height of 1.5–2.7 kJ/mol depending on the membrane: lowest for DPPG and highest for DPPC. This is followed by a pronounced stable state at the inner site of the headgroups, between 1.0 and 1.5 nm from the center. This free energy minimum is similarly deep for DPPC, DPPG, and DPPA (–5.9 to –5.1 kJ/mol), but notably shallower in DPPE, at –3.4 kJ/mol. This is the global minimum for all membranes. Toward the bilayer center, a central barrier of 1.2–2.2 kJ/mol is observed, with the lowest value for DPPA and the highest for DPPG.

In the cases of DPPA and DPPE membranes, an additional free energy minimum appears at the bilayer center for DIOX and OXA, and to a lesser extent for PIR. Similar profiles have been reported for amphiphilic molecules like ethanol or isopropanol [70], the hydrophobic hexane [71], and nanoparticle models [72] where the bilayer center acts as a shallow energetic trap due to increased free volume and disorder. This effect is more pronounced in thicker, more ordered bilayers, where the dense acyl chain region near the headgroups restricts solute accommodation. A similar link between membrane thickness and barrier width was shown in systems with increasing lipid tail length, where thicker membranes exhibited broader and slightly higher free energy barriers with similar metastable state at the very center [21]—trends also observed for DPPA and DPPE compared to the thinner bilayers, DPPC and DPPG.

Free energy profiles obtained using the AWH method showed good agreement with profiles calculated from density distributions (see Supplementary Materials, Figure S3), confirming the reliability of the results.

### 2.3. Concentration Dependence of Membrane Penetration

**Figure 6:**
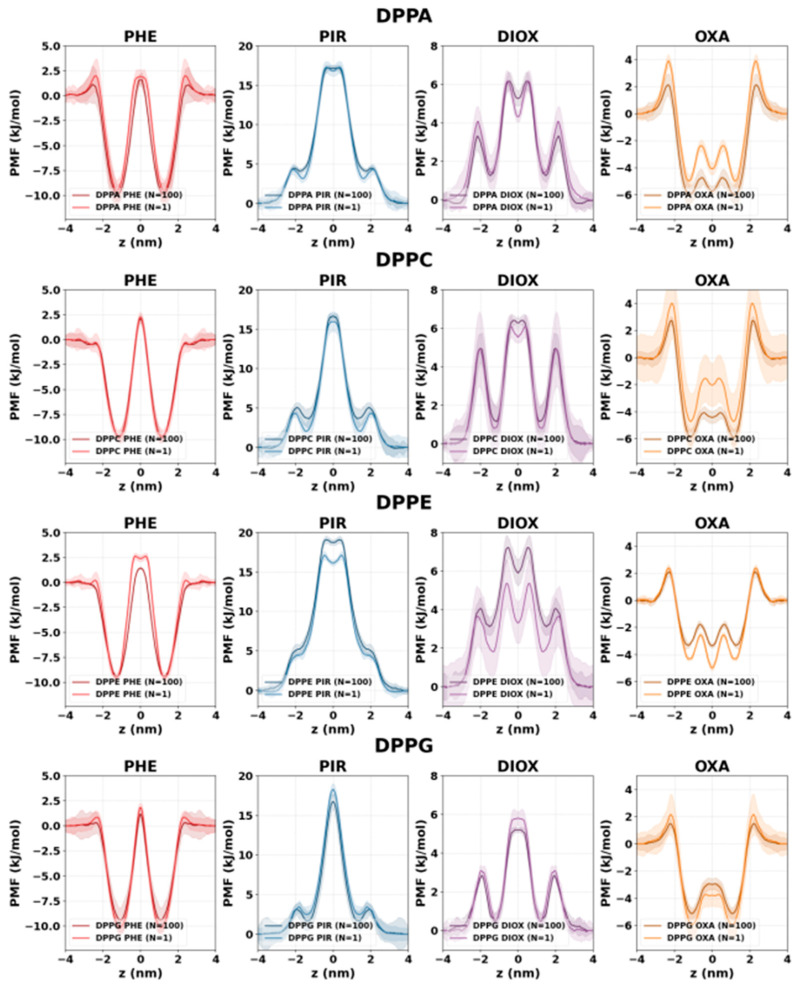
Free energy profiles of the investigated compounds in different phospholipid membranes, calculated by AWH at infinite dilution (N = 1, low concentration; lighter color) and at high concentration (darker color). Results were obtained by molecular dynamics simulations performed in this study using GROMACS 2023.2 and the CHARMM force field.

The concentration dependence of membrane penetration (as shown in Figure 6) was evaluated by comparing free energy profiles obtained with the AWH method at infinite dilution, with only a single NSO-HET molecule (N = 1) present and at previously discussed high concentrations. Generally, only minor differences appeared when membrane structural changes were minimal, as in the cases of DPPC and DPPG.

In contrast, in the case of DPPE membranes, where high concentrations disrupt lipid tail ordering and reduce membrane thickness, more noticeable differences arise. For phenol, the central barrier is about 1.8 kJ/mol higher and wider (~0.6 nm) at infinite dilution, reflecting a more ordered bilayer. Conversely, for DIOX and PIR, the central barrier is lower by 1.3 kJ/mol and 0.5 kJ/mol, respectively, at infinite dilution. OXA behaves differently: its central free energy minimum is 1.63 kJ/mol deeper at infinite dilution, indicating a thermodynamically more favorable position without membrane disruption.

While DPPA membranes also have an initially more ordered structure, concentration has less effect on the PMF profiles than in DPPE membranes. At the headgroups region, where there is a large amount of charge, the free energy barrier is slightly higher at infinite dilution for almost all pollutants. This can be linked to the permeant accumulation near the headgroups, increasing APL at higher concentrations and lowering the energy barrier for entry.

For PIR, the height of the central barrier is affected only in DPPE membranes. However, the inner metastable state near the headgroup region shows subtle concentration-dependent changes across all membranes, being slightly higher (by less than 1 kJ/mol) and less defined at higher concentrations. For DIOX, concentration does not affect the outer barrier at the headgroups in DPPG and DPPC membranes, but it is slightly higher in DPPA (~0.6 kJ/mol) and lower in DPPE, as discussed earlier.

The PMF profile of OXA shows the most pronounced concentration dependence. The outer barrier at the headgroups is higher at infinite dilution by 1.3 kJ/mol in DPPC and 1.8 kJ/mol in DPPA. The depth of the free energy minimum at the membrane center is affected in all membranes: it is highest in DPPC, where at infinite dilution the central metastable state is 2.4 kJ/mol higher in free energy, while in DPPE the central minimum is 1.6 kJ/mol lower at infinite dilution, as noted earlier.

## 3. Materials and Methods

### 3.1. System Preparation

To systematically analyze how individual phospholipid species influence the interaction and permeation behavior of small, potentially toxic molecules, twenty distinct membrane systems were simulated. Each system was composed of a homogeneous bilayer containing only one lipid type—DPPC, DPPE, DPPG, or DPPA—allowing for direct comparison of lipid-specific effects. For each lipid type, separate simulations were performed in the presence of a single NSO-HET compound—*N*-methyl-2-pyrrolidone, 1,4-dioxane, oxane, or phenol—resulting in a total of sixteen systems with NSO-HET exposure. Additionally, one pure bilayer system was simulated for each lipid type as a reference, yielding four control systems.

In the cases of DPPE and DPPA, where the phase transition temperatures are higher (337 K [6,60] and 339 K [58,59] respectively), simulations were performed at elevated temperatures. This set of simulations enabled a detailed investigation into how small molecule presence modulates membrane structure and organization in a lipid headgroup-specific manner. A summary of all the membrane systems simulated, along with their referred names and conditions, is presented in Table 2.

Initial membrane configurations were generated using CHARMM-GUI’s [73] Membrane Builder [74,75], each containing 128 lipid molecules per leaflet (n_lipid_ = 256) and 50 water molecules per lipid headgroup (n_water_ = 12,800). The membrane dimensions were selected to be sufficiently large to reproduce relevant membrane structural parameters, as demonstrated in previous studies [76,77,78,79]. An ion concentration of 150 mM NaCl was used, and in the cases of charged phospholipids, sodium was used as a counterion. In addition, 100 NSO-HET molecules were randomly inserted into each simulation box, resulting in a concentration of 0.432 M. This elevated concentration was intentionally chosen to amplify relevant structural changes and to distinguish them from statistical fluctuations [80].

The CHARMM36 [81,82] force field was used for the phospholipids, while water molecules were represented using the CHARMM implementation of the TIP3P [83] model. NSO-HET molecules were parameterized using the CGenFF force field [84]. Periodic boundary conditions were applied in all three spatial dimensions of the cubic simulation box. Molecular dynamics (MD) simulations were performed using GROMACS 2023.2, and molecular visualizations were conducted with VMD 1.9.3 [85].

### 3.2. Parameters of MD Simulations

The simulation protocol outlined by CHARMM-GUI was adopted with slight modifications to prepare the simulations [74,75,86]. This included energy minimization via the steepest descent algorithm, where the maximum number of steps was set to 5000, which was followed by a six-step equilibration procedure in which positional restraints on the phosphorus atoms of the lipid molecules were gradually reduced from 1000 kJ/(mol·nm^2^) to prevent decomposition of the bilayer [81]. In the first three equilibration steps, the equations of motion were integrated with a timestep of 1 fs, while a 2 fs timestep was used in all subsequent stages. NVT ensemble was used during the first two equilibration steps, followed by NPT ensemble for the remaining ones. Each equilibration stage was 125 ps long, except for the sixth stage, which lasted 10 ns. Throughout equilibration, the Berendsen thermostat and barostat [87] were employed, with coupling constants τ_T_ = 1.0 ps and τ_P_ = 5.0 ps, respectively. All systems were simulated at either 330 K (in the cases of DPPC and DPPG) or 345 K (in the cases of DPPA and DPPE) and at 1 atm.

During the production runs, the Nosé–Hoover thermostat and the Parrinello–Rahman barostat were used [88,89,90]. Temperature, pressure, and coupling constants were kept consistent with those applied during the NPT equilibration stages. Heavy atom–hydrogen distances were constrained using the P-LINCS algorithm [91] and water molecules were kept rigid using SETTLE [92]. A switch function starting at 10 Å was used for the van der Waals (vdW) interactions with a cutoff of 12 Å. The Particle–Mesh Ewald (PME) algorithm [93] was used to calculate the long-range electrostatic interactions with a short-range cutoff set also at 12 Å.

Production runs of 500 ns were carried out for each system, resulting in a total of 20 simulations and 10 μs of classical MD simulation time. The stability of each simulation was monitored by tracking changes in the box dimensions (see Supplementary Materials, Figure S1). Based on this analysis, the first 200 ns were excluded from evaluation to ensure that only equilibrated states were considered. The remaining 300 ns of each trajectory were used for all subsequent analyses.

### 3.3. Analysis of Structural Membrane Parameters

Structural membrane parameters area per lipid (APL), volume per lipid (VPL), deuterium order parameter (S_CD_), and membrane thickness (D_HH_) were analyzed to understand the structural changes induced on the different phospholipid membranes by the investigated NSO-HET molecules. The definitions and the equations are listed in Table 3.

### 3.4. Accuracy of the Simulation Protocol

To evaluate the accuracy of the simulations, structural membrane parameters obtained from the pollutant-free (without NSO-HET) systems were compared with available literature data for all four lipid types investigated (DPPC, DPPA, DPPE, and DPPG). Key parameters such as area per lipid (APL), membrane thickness (D_HH_), and volume per lipid (VPL) were used for validation. The results, summarized in Table 4, show good agreement with reported experimental values.

The largest deviations were observed for DPPE and DPPA, which are less extensively discussed in the literature. Overall, the consistency between the simulated and experimental data supports the reliability of the CHARMM36 force field and the applied simulation protocol in reproducing the structural features of lipid bilayers.

### 3.5. Free Energy Calculations

To compute the potential of mean force (PMF) associated with the membrane permeation of NSO-HET molecules, the Accelerated Weight Histogram (AWH) method implemented in GROMACS was employed. The one-dimensional reaction coordinate (z) was defined as the distance along the membrane normal between the center of mass of the penetrating NSO-HET molecule and that of the lipid bilayer.

Like other adaptive biasing techniques [27,28,100,101], a history-dependent biasing potential was added to the system in an adaptive fashion to overcome free energy barriers along the reaction coordinate and to achieve a flat target distribution. AWH requires only a few input parameters for reliable estimation of PMF profiles, which are relatively insensitive to the specific identity of the permeant molecule [32,34].

Initial configurations for the AWH simulations were extracted from unbiased trajectories, selecting frames in which the NSO-HET molecules were in the bulk aqueous phase, outside the membrane. The initial update size of the free energy in the AWH simulations were controlled by setting an estimate of the diffusion constant along the reaction coordinate (2.5 × 10^−4^ nm^2^ ps^−1^) together with an initial error estimate (10 kJ mol^−1^). These parameters influence the rate at which the bias adapts and affect convergence speed, but do not impact the final PMF profile [35]. The harmonic pull potential for the AWH force constant was set as 12,800 kJ mol^−1^ nm^−2^. All other simulation parameters were identical to those used in the classical MD simulations.

Each AWH simulation was run for a minimum of 1.2 μs, with extensions up to 3 μs when convergence was not reached. For each permeant–membrane system, at least two independent simulations were conducted starting from different initial configurations. In cases where convergence was insufficient, two additional replicates were performed. After completion, individual PMF profiles were symmetrized and the average and standard deviation of the data were calculated.

The PMF profiles were also calculated from the density profiles ρ(*z*) obtained from the classical MD simulations using the inverse Boltzmann formula with the aqueous bulk density of NSO-HET molecules (ρ_0_) as reference [102,103]:

$$PMF(z)=-RTln\frac{\rho (z)}{\rho_{0}}$$

## 4. Conclusions

The passive membrane penetration of NSO-HET compounds is governed by interplay between solute polarity and membrane composition, the latter being directly linked to the bilayer’s structural properties. While all tested solutes were able to penetrate the model lipid bilayers, their concentration within the membrane, insertion depth, energetic cost of penetration, and structural impact were all modulated by the chemical identity of the phospholipid headgroups.

Across all membrane types, DPPG consistently supports the highest membrane partitioning, as reflected in the logP_memb/bulk_ values. Compared to DPPE, DPPG increases membrane affinity by 27–63% depending on the compound, with PHE showing the smallest increase of 27.5% and DIOX the largest at 63.7%. This effect is rooted in the larger, polar, and negatively charged glycerol headgroup of DPPG, which results in less tight packing and less ordered lipid tails, thereby supporting greater permeability. In contrast, DPPE, with its small, compact, and zwitterionic ethanolamine headgroup, presents the lowest permeability barrier due to its tight lipid packing and reduced hydration.

Free energy profiles of membrane penetration further support this trend. Although all individual solutes exhibit similar profile shapes, characterized by barriers at the headgroup region and the bilayer center, the heights and widths of these barriers vary depending on membrane type. For instance, the central barrier for PIR is 19.2 kJ/mol in DPPE and 16.6 kJ/mol in DPPG, representing a 13.4% reduction in DPPG, and indicating a lower energetic cost of penetration. Similarly, DIOX shows a central barrier of 7.2 kJ/mol in DPPE, which decreases to 5.2 kJ/mol in DPPG—a 27.4% drop, reinforcing the permeability advantage of DPPG. For OXA, the central barrier remains low across all membranes (1.2–2.2 kJ/mol); however, its interfacial free energy minimum is notably shallower in DPPE (−3.4 kJ/mol) compared to −5.9 kJ/mol in DPPC or DPPG—equivalent to a 43.2% reduction in stabilization energy at this location. Phenol, the most lipophilic solute in the set, shows deep interfacial free energy wells and a relatively uniform central barrier (10.6–12.0 kJ/mol) across membrane types.

These energetic trends align with solute density distributions and structural perturbation data. PIR and DIOX accumulate at lower concentrations within the bilayers, localizing primarily at the headgroup region, and induce only modest structural changes. Their limited penetration is consistent with both their low logP_memb/bulk_ values and the high central barriers observed in their PMF profiles. In contrast, OXA and PHE accumulate at higher concentrations within the membrane and cause more pronounced structural disturbances, including reductions in bilayer thickness and lipid tail order. These effects are most pronounced in DPPA and DPPE membranes, where the acyl chains are initially highly ordered and the membrane thickness is higher. Among all studied compounds, OXA exhibits the most distinct density profile, showing a higher concentration in the bilayer center than in the bulk aqueous phase. This behavior is consistent with its free energy profiles, which indicate that the membrane center is thermodynamically preferred over bulk water, with a free energy difference of −3.4 to −5.9 kJ/mol. In the cases of OXA and DIOX, an additional free energy minimum is found at the bilayer center in DPPA and DPPE membranes, a feature associated with increased bilayer thickness and hydrophobic core stability. These shallow energetic traps, previously observed for amphiphilic alcohols and nanoparticles, may act as transient solute binding sites, particularly in more ordered membranes.

The concentration dependence of the membrane penetration of NSO-HETs was also investigated. In general, changes in free energy profiles between simulations at infinite dilution and at high concentrations are modest in DPPC and DPPG membranes, where bilayer structure is less affected by pollutant accumulation. However, in DPPE, where high pollutant loading significantly disrupts acyl chain ordering, the impact is more pronounced. For example, the phenol’s central barrier increases by 1.8 kJ/mol and broadens by 0.6 nm at infinite dilution, reflecting a more ordered and less permeable bilayer structure in the absence of structural perturbation. Conversely, for OXA, the free energy minimum near the bilayer center becomes 1.6 kJ/mol deeper at infinite dilution in DPPE, indicating a more favorable thermodynamic state without membrane disruption. DIOX and PIR show similar trends, with lower central barriers at infinite dilution in DPPE, but minimal differences in DPPC or DPPG. These results confirm that concentration-dependent effects are membrane-specific and most pronounced in bilayers that are initially more compact and ordered.

In summary, the data demonstrated that different phospholipid headgroups modulate solute permeability through a combination of thermodynamic and structural mechanisms. DPPG, with its less tightly packed headgroups and more disordered inner structure, supports higher permeability and accommodates solute insertion with minimal structural disruption. In contrast, DPPE offers both thermodynamic and physical resistance to additive uptake. These findings underscore the importance of headgroup polarity, size, and charge in shaping both the penetration energetics and biophysical consequences of NSO-HET compounds on lipid membranes.

## Figures and Tables

**Table 1:** Calculated logP_memb/bulk_ of the investigated membrane systems. Results were obtained by molecular dynamics simulations performed in this study using GROMACS 2023.2 and the CHARMM force field.

LogP_memb/bulk_	DPPA	DPPC	DPPE	DPPG
PHE	0.89	0.93	0.72	0.99
PIR	−0.87	−0.71	−1.08	−0.54
DIOX	−0.37	−0.31	−0.55	−0.20
OXA	0.67	0.75	0.54	0.79

**Table 2:** Names, compositions, and simulation temperatures of the simulated membrane systems.

Name	Phospholipid	NSO-HET	T (K)
DPPC	DPPC	-	330
DPPC-DIOX	DPPC	1,4-Dioxane	330
DPPC-OXA	DPPC	Oxane	330
DPPC-PHE	DPPC	Phenol	330
DPPC-PIR	DPPC	N-Methyl-2-Pyrrolidone	330
DPPG	DPPG	-	330
DPPG-DIOX	DPPG	1,4-Dioxane	330
DPPG-OXA	DPPG	Oxane	330
DPPG-PHE	DPPG	Phenol	330
DPPG-PIR	DPPG	N-Methyl-2-Pyrrolidone	330
DPPE	DPPE	-	345
DPPE-DIOX	DPPE	1,4-Dioxane	345
DPPE-OXA	DPPE	Oxane	345
DPPE-PHE	DPPE	Phenol	345
DPPE-PIR	DPPE	N-Methyl-2-Pyrrolidone	345
DPPA	DPPA	-	345
DPPA-DIOX	DPPA	1,4-Dioxane	345
DPPA-OXA	DPPA	Oxane	345
DPPA-PHE	DPPA	Phenol	345
DPPA-PIR	DPPA	N-Methyl-2-Pyrrolidone	345

**Table 3:** Definition of the calculated membrane parameters.

Membrane Parameter	Definition	Formula	Abbreviations
APL	Area per Lipid [94]	$\frac{2\times L_{x}\times L_{y}}{n_{lipid}}$	L: average x and y dimensions of the simulation box
D**_HH_**	Membrane Thickness [95]	Distance between peaks in the membrane electron density profile	-
VPL	Volume per Lipid [94]	$\frac{D_{HH}\times APL}{2}$	-
S**_CD_**	Deuterium Order Parameter, measures the orientation of C-H bonds with respect to the bilayer normal [95]	$\frac{1}{2}\langle 3{cos}^{2}\Theta -1\rangle$	Θ: the angle between bilayer normal (Z) and the vector between C_i_-H_i_

**Table 4:** Comparison of the calculated membrane parameters with data from the literature. Experimental values (exp) are from the literature, while simulated values (sim) were obtained via molecular dynamics simulations using GROMACS 2023.2 and CHARMM force field (see Section 3). In each cell, the average value is given in bold, with the associated error on the following line. Temperatures from the literature sources are indicated in italics.

Membrane Parameter	DPPC (exp)	DPPC (sim) T = 330 K	DPPE (exp)	DPPE (sim) T = 345 K	DPPA (exp)	DPPA (sim) T = 345 K	DPPG (exp)	DPPG (sim) T = 330 K
APL (nm^2^)	**0.621** ± 0.02 *(323.15 K)* [96]	**0.618** ± 0.014	**0.605** *(342.15 K)* [97] **0.610** ± 0.32 *(342.15 K)* [12]	**0.562** ± 0.009	**0.393** ± 0.13 *(298.15 K)* [16] **~0.42** *(298.15 K)* [98]	**0.581** ± 0.010	**0.670** * (323.15 K)* [5]	**0.654** ± 0.013
D_HH_ (nm)	**3.850** *(323.15 K)* [96]	**3.732** ± 0.25	**4.100** ± 0.10 *(343.15 K)* [99]	**4.059** ± 0.31	**4.640** ± 0.02 *(303.15 K)* [16]	**3.828** ± 0.29	**3.550** *(323.15 K)* [5]	**3.495** ± 0.27
VPL (nm^3^)	**1.232 ** *(323.15 K)* [96]	**1.152** ± 0.02	-	**1.141** ± 0.05	-	**1.111** ± 0.06	**1.189** *(323.15 K)* [5]	**1.143** ± 0.03

## Data Availability

The data presented in this study are available on request from the corresponding author. The data are not publicly available due to the policy of the University of Miskolc.

## Supplementary Materials

The following supporting information can be downloaded at: https://www.mdpi.com/article/10.3390/ijms26157427/s1.

## References

[1] Palaiokostas M., Ding W., Shahane G., Orsi M. (2018). Effects of Lipid Composition on Membrane Permeation. Soft Matter.

[2] Pogozheva I.D., Armstrong G.A., Kong L., Hartnagel T.J., Carpino C.A., Gee S.E., Picarello D.M., Rubin A.S., Lee J., Park S. (2022). Comparative Molecular Dynamics Simulation Studies of Realistic Eukaryotic, Prokaryotic, and Archaeal Membranes. J. Chem. Inf. Model..

[3] Santamaria A., Batchu K.C., Fragneto G., Laux V., Haertlein M., Darwish T.A., Russell R.A., Zaccai N.R., Guzmán E., Maestro A. (2023). Investigation on the Relationship between Lipid Composition and Structure in Model Membranes Composed of Extracted Natural Phospholipids. J. Colloid Interface Sci..

[4] Dowhan W. (1997). Molecular Basis for Membrane Phospholipid Diversity: Why Are There So Many Lipids?. Annu. Rev. Biochem..

[5] Pan J., Heberle F.A., Tristram-Nagle S., Szymanski M., Koepfinger M., Katsaras J., Kučerka N. (2012). Molecular Structures of Fluid Phase Phosphatidylglycerol Bilayers as Determined by Small Angle Neutron and X-Ray Scattering. Biochim. Biophys. Acta–Biomembr..

[6] Leekumjorn S., Sum A.K. (2006). Molecular Simulation Study of Structural and Dynamic Properties of Mixed DPPC/DPPE Bilayers. Biophys. J..

[7] Zhukov A., Popov V. (2023). Eukaryotic Cell Membranes: Structure, Composition, Research Methods and Computational Modelling. Int. J. Mol. Sci..

[8] Uran S., Larsen Å., Jacobsen P.B., Skotland T. (2001). Analysis of Phospholipid Species in Human Blood Using Normal-Phase Liquid Chromatography Coupled with Electrospray Ionization Ion-Trap Tandem Mass Spectrometry. J. Chromatogr. B Biomed. Sci. Appl..

[9] Leekumjorn S., Sum A.K. (2007). Molecular Studies of the Gel to Liquid-Crystalline Phase Transition for Fully Hydrated DPPC and DPPE Bilayers. Biochim. Biophys. Acta–Biomembr..

[10] Jahn R., Grubmüller H. (2002). Membrane Fusion. Curr. Opin. Cell Biol..

[11] Goetz G.J., Naomi S., Madrigal A.M., Chang C.L.A., Cornell C.E., Keller S.L. (2025). Micron-Scale, Liquid-Liquid Phase Separation in Ternary Lipid Membranes Containing DPPE. bioRXiv.

[12] Molugu T.R., Thurmond R.L., Alam T.M., Trouard T.P., Brown M.F. (2022). Phospholipid Headgroups Govern Area per Lipid and Emergent Elastic Properties of Bilayers. Biophys. J..

[13] Uppulury K., Coppock P.S., Kindt J.T. (2015). Molecular Simulation of the DPPE Lipid Bilayer Gel Phase: Coupling between Molecular Packing Order and Tail Tilt Angle. J. Phys. Chem. B.

[14] Maleš P., Butumović M., Erceg I., Brkljača Z., Bakarić D. (2023). Influence of DPPE Surface Undulations on Melting Temperature Determination: UV/Vis Spectroscopic and MD Study. Biochim. Biophys. Acta–Biomembr..

[15] Kobayashi K., Jimbo H., Nakamura Y., Wada H. (2024). Biosynthesis of Phosphatidylglycerol in Photosynthetic Organisms. Prog. Lipid Res..

[16] Drabik D., Czogalla A. (2021). Simple Does Not Mean Trivial: Behavior of Phosphatidic Acid in Lipid Mono- and Bilayers. Int. J. Mol. Sci..

[17] Shinoda W. (2016). Permeability across Lipid Membranes. Biochim. Biophys. Acta–Biomembr..

[18] Benmameri M., Chantemargue B., Humeau A., Trouillas P., Fabre G. (2023). MemCross: Accelerated Weight Histogram Method to Assess Membrane Permeability. Biochim. Biophys. Acta–Biomembr..

[19] Venable R.M., Krämer A., Pastor R.W. (2019). Molecular Dynamics Simulations of Membrane Permeability. Chem. Rev..

[20] Vermaas J.V., Dixon R.A., Chen F., Mansfield S.D., Boerjan W., Ralph J., Crowley M.F., Beckham G.T. (2019). Passive Membrane Transport of Lignin-Related Compounds. Proc. Natl. Acad. Sci. USA.

[21] Frallicciardi J., Melcr J., Siginou P., Marrink S.J., Poolman B. (2022). Membrane Thickness, Lipid Phase and Sterol Type Are Determining Factors in the Permeability of Membranes to Small Solutes. Nat. Commun..

[22] Koyanagi T., Leriche G., Yep A., Onofrei D., Holland G.P., Mayer M., Yang J. (2016). Effect of Headgroups on Small-Ion Permeability across Archaea-Inspired Tetraether Lipid Membranes. Chem. Eur. J..

[23] DeMarco K.R., Bekker S., Clancy C.E., Noskov S.Y., Vorobyov I. (2018). Digging into Lipid Membrane Permeation for Cardiac Ion Channel Blocker D-Sotalol with All-Atom Simulations. Front. Pharmacol..

[24] Ingólfsson H.I., Arnarez C., Periole X., Marrink S.J. (2016). Computational ‘Microscopy’ of Cellular Membranes. J. Cell Sci..

[25] Javanainen M., Martinez-Seara H., Vattulainen I. (2017). Excessive Aggregation of Membrane Proteins in the Martini Model. PLoS ONE.

[26] Torrie G., Valleau J. (1977). Nonphysical Sampling Distributions in Monte Carlo Free-Energy Estimation: Umbrella Sampling. J. Comput. Phys..

[27] Comer J., Gumbart J.C., Hénin J., Lelièvre T., Pohorille A., Chipot C. (2015). The Adaptive Biasing Force Method: Everything You Always Wanted to Know but Were Afraid to Ask. J. Phys. Chem. B.

[28] Darve E., Rodríguez-Gómez D., Pohorille A. (2008). Adaptive Biasing Force Method for Scalar and Vector Free Energy Calculations. J. Chem. Phys..

[29] Tse C.H., Comer J., Chu S.K.S., Wang Y., Chipot C. (2019). Affordable Membrane Permeability Calculations: Permeation of Short-Chain Alcohols through Pure-Lipid Bilayers and a Mammalian Cell Membrane. J. Chem. Theory Comput..

[30] Laio A., Parrinello M. (2002). Escaping Free-Energy Minima. Proc. Natl. Acad. Sci. USA.

[31] Lindahl V., Lidmar J., Hess B. (2018). Riemann Metric Approach to Optimal Sampling of Multidimensional Free-Energy Landscapes. Phys. Rev. E.

[32] Lindahl V., Villa A., Hess B. (2017). Sequence Dependency of Canonical Base Pair Opening in the DNA Double Helix. PLoS Comput. Biol..

[33] Lindahl V., Gourdon P., Andersson M., Hess B. (2018). Permeability and Ammonia Selectivity in Aquaporin TIP2;1: Linking Structure to Function. Sci. Rep..

[34] Lindahl V., Lidmar J., Hess B. (2014). Accelerated Weight Histogram Method for Exploring Free Energy Landscapes. J. Chem. Phys..

[35] Lundborg M., Wennberg C., Lidmar J., Hess B., Lindahl E., Norlén L. (2022). Skin Permeability Prediction with MD Simulation Sampling Spatial and Alchemical Reaction Coordinates. Biophys. J..

[36] Brinkmann M., Schneider A.L., Bluhm K., Schiwy S., Lehmann G., Deutschmann B., Müller A., Tiehm A., Hollert H. (2019). Ecotoxicity of Nitrogen, Sulfur, or Oxygen Heterocycles and Short-Chained Alkyl Phenols Commonly Detected in Contaminated Groundwater. Environ. Toxicol. Chem..

[37] Sikkema J., de Bont J.A., Poolman B. (1994). Interactions of Cyclic Hydrocarbons with Biological Membranes. J. Biol. Chem..

[38] Sikkema J., de Bont J.A., Poolman B. (1995). Mechanisms of Membrane Toxicity of Hydrocarbons. Microbiol. Rev..

[39] De Smet M.J., Jaap K., Witholt B. (1978). The Effect of Toluene on the Structure and Permeability of the Outer and Cytoplasmic Membranes of Escherichia coli. Biochim. Biophys. Acta–Biomembr..

[40] Uribe S., Rangel P., Espinola G., Aguirre G. (1990). Effects of Cyclohexane, an Industrial Solvent, on the Yeast Saccharomyces cerevisiae and on Isolated Yeast Mitochondria. Appl. Environ. Microbiol..

[41] Uribe S., Ramirez J., Pena A. (1985). Effects of β-Pinene on Yeast Membrane Functions. J. Bacteriol..

[42] Raz A., Livne A. (1973). Differential Effects of Lipids on the Osmotic Fragility of Erythrocytes. Biochim. Biophys. Acta–Biomembr..

[43] Seeman P. (1972). The Membrane Actions of Anesthetics and Tranquilizers. Pharmacol. Rev..

[44] Shaikh M.S.I., Derle N.D., Bhamber R. (2012). Permeability Enhancement Techniques for Poorly Permeable Drugs: A Review. J. Appl. Pharm. Sci..

[45] Yang L., Zhang H., Mikov M., Tucker I.G. (2009). Physicochemical and Biological Characterization of Monoketocholic Acid, a Novel Permeability Enhancer. Mol. Pharm..

[46] Gurtovenko A.A., Anwar J., Yorkshire W. (2007). Modulating the Structure and Properties of Cell Membranes: The Molecular Mechanism of Action of Dimethyl Sulfoxide. J. Phys. Chem. B.

[47] Robbiano L., Baroni D., Carrozzino R., Mereto E., Brambilla G. (2004). DNA Damage and Micronuclei Induced in Rat and Human Kidney Cells by Six Chemicals Carcinogenic to the Rat Kidney. Toxicology.

[48] Blotevogel J., Reineke A.-K., Hollender J., Held T. (2008). Identifikation NSO-Heterocyclischer Prioritärsubstanzen zur Erkundung und Überwachung Teeröl-Kontaminierter Standorte. Grundwasser.

[49] Wang J., Chi Q., Zhang R., Wu X., Jiang X., Mu Y., Tu Y., Shen J. (2022). Evaluation of N-Methylpyrrolidone Bio-Mineralization Mechanism and Bacterial Community Evolution under Denitrification Environment. J. Clean. Prod..

[50] Adil N., Jamil Z., Iqbal J., Siddiqui A.J., Sibt-e-Hassan S., Kumari S., Ali S.A., Musharraf S.G. (2025). The Pesticide Paradox: Metabolomics Insights into N-Methyl-2-Pyrrolidone (NMP) Exposure as a Culprit of Infant Undernourishment. J. Hazard. Mater. Adv..

[51] Gao Y., Chen T., Hou Y., Xue R., Liu R., Chen F., Zhang Y., Rittmann B.E. (2023). The Roles of *Methylobacterium organophilum* and *Sphingomonas melonis* for Accelerating N-Methyl Pyrrolidone (NMP) Biodegradation. J. Water Process Eng..

[52] Ginsberg G., Chen Y., Vasiliou V. (2022). Mechanistic Considerations in 1,4-Dioxane Cancer Risk Assessment. Curr. Opin. Environ. Sci. Health.

[53] Rafat M., Ghazy M.A., Nasr M. (2024). Phycoremediation of 1,4 Dioxane-Laden Wastewater: A Techno-Economic and Sustainable Development Approach. J. Environ. Manag..

[54] Dastidar R.G., Kim M.S., Zhou P., Luo Z., Shi C., Barnett K.J., McClelland D.J., Chen E.Y.-X., Van Lehn R.C., Huber G.W. (2022). Catalytic Production of Tetrahydropyran (THP): A Biomass-Derived, Economically Competitive Solvent with Demonstrated Use in Plastic Dissolution. Green Chem..

[55] Hartness S.W., Saab M., Preußker M., Mazzotta R., Dewey N.S., Hill A.W., Vanhove G., Fenard Y., Heufer K.A., Rotavera B. (2024). Low-Temperature Ignition and Oxidation Mechanisms of Tetrahydropyran. Proc. Combust. Inst..

[56] Mahgoub S.A., Qattan S.Y.A., Salem S.S., Abdelbasit H.M., Raafat M., Ashkan M.F., Al-Quwaie D.A., Motwali E.A., Alqahtani F.S., Abd El-Fattah H.I. (2023). Characterization and Biodegradation of Phenol by Pseudomonas aeruginosa and *Klebsiella variicola* Strains Isolated from Sewage Sludge and Their Effect on Soybean Seeds Germination. Molecules.

[57] Panigrahy N., Priyadarshini A., Sahoo M.M., Verma A.K., Daverey A., Sahoo N.K. (2022). A Comprehensive Review on Eco-Toxicity and Biodegradation of Phenolics: Recent Progress and Future Outlook. Environ. Technol. Innov..

[58] Khvedelidze M., Mdzinarashvili T., Shekiladze E., Schneider M., Moersdorf D., Bernhardt I. (2015). Structure of Drug Delivery DPPA and DPPC Liposomes with Ligands and Their Permeability through Cells. J. Liposome Res..

[59] Wang H.-Y., Tümmler B., Boggs J.M. (1989). Use of Spin Labels to Determine the Percentage of Interdigitated Lipid in Complexes with Polymyxin B and Polymyxin B Nonapeptide. Biochim. Biophys. Acta–Biomembr..

[60] Petrov A.G., Gawrisch K., Brezesinski G., Klose G., Möps A. (1982). Optical Detection of Phase Transitions in Simple and Mixed Lipid–Water Phases. Biochim. Biophys. Acta–Biomembr..

[61] Arvayo-Zatarain J.A., Favela-Rosales F., Contreras-Aburto C., Urrutia-Bañuelos E., Maldonado A. (2019). Molecular Dynamics Simulation Study of the Effect of Halothane on Mixed DPPC/DPPE Phospholipid Membranes. J. Mol. Model..

[62] Rózsa Z.B., Szőri-Dorogházi E., Viskolcz B., Szőri M. (2021). Transmembrane Penetration Mechanism of Cyclic Pollutants Inspected by Molecular Dynamics and Metadynamics: The Case of Morpholine, Phenol, 1,4-Dioxane and Oxane. Phys. Chem. Chem. Phys..

[63] Machleidt H., Roth S., Seeman P. (1972). The Hydrophobic Expansion of Erythrocyte Membranes by the Phenol Anesthetics. Biochim. Biophys. Acta–Biomembr..

[64] Martin Y.C. (1996). Exploring QSAR: Hydrophobic, Electronic, and Steric Constants. J. Med. Chem..

[65] Cumming H., Rücker C. (2017). Octanol–Water Partition Coefficient Measurement by a Simple ^1^H NMR Method. ACS Omega.

[66] Sasaki H., Kojima M., Mori Y., Nakamura J., Shibasaki J. (1990). Enhancing Effect of Pyrrolidone Derivatives on Transdermal Drug Delivery II. Effect of Application Concentration and Pre-Treatment of Enhancer. Int. J. Pharm..

[67] Shafer W.E., Schönherr J. (1985). Accumulation and transport of phenol, 2-nitrophenol, and 4-nitrophenol in plant cuticles. Ecotoxicol. Environ. Saf..

[68] Ingram T., Storm S., Kloss L., Mehling T., Jakobtorweihen S., Smirnova I. (2013). Prediction of micelle/water and liposome/water partition coefficients based on molecular dynamics simulations, COSMO-RS, and COSMOmic. Langmuir.

[69] Marrink S.J., Berendsen H.J.C. (1996). Permeation process of small molecules across lipid membranes studied by molecular dynamics simulations. J. Phys. Chem..

[70] Wang Y., Gallagher E., Jorgensen C., Troendle E.P., Hu D., Searson P.C., Ulmschneider M.B. (2019). An experimentally validated approach to calculate the blood-brain barrier permeability of small molecules. Sci. Rep..

[71] MacCallum J.L., Tieleman D.P. (2006). Computer simulation of the distribution of hexane in a lipid bilayer: Spatially resolved free energy, entropy, and enthalpy profiles. J. Am. Chem. Soc..

[72] Su C.-F., Merlitz H., Rabbel H., Sommer J.-U. (2017). Nanoparticles of various degrees of hydrophobicity interacting with lipid membranes. J. Phys. Chem. Lett..

[73] Lee J., Cheng X., Swails J.M., Yeom M.S., Eastman P.K., Lemkul J.A., Wei S., Buckner J., Jeong J.C., Qi Y. (2016). CHARMM-GUI input generator for NAMD, GROMACS, AMBER, OpenMM, and CHARMM/OpenMM simulations using the CHARMM36 additive force field. J. Chem. Theory Comput..

[74] Lee J., Patel D.S., Ståhle J., Park S.-J., Kern N.R., Kim S., Lee J., Cheng X., Valvano M.A., Holst O. (2019). CHARMM-GUI membrane builder for complex biological membrane simulations with glycolipids and lipoglycans. J. Chem. Theory Comput..

[75] Wu E.L., Cheng X., Jo S., Rui H., Song K.C., Dávila-Contreras E.M., Qi Y., Lee J., Monje-Galvan V., Venable R.M. (2014). CHARMM-GUI membrane builder—Toward realistic biological membrane simulations. J. Comput. Chem..

[76] Kumar N., Sastry G.N. (2021). Study of lipid heterogeneity on bilayer membranes using molecular dynamics simulations. J. Mol. Graph. Model..

[77] Wang W., Zhang J., Qiu Z., Cui Z., Li N., Li X., Wang Y., Zhang H., Zhao C. (2022). Effects of polyethylene microplastics on cell membranes: A combined study of experiments and molecular dynamics simulations. J. Hazard. Mater..

[78] Talandashti R., Mehrnejad F., Rostamipour K., Doustdar F., Lavasanifar A. (2021). Molecular insights into pore formation mechanism, membrane perturbation, and water permeation by the antimicrobial peptide pleurocidin: A combined all-atom and coarse-grained molecular dynamics simulation study. J. Phys. Chem. B.

[79] Hu Y., Patel S. (2016). Thermodynamics of cell-penetrating HIV1 TAT peptide insertion into PC/PS/CHOL model bilayers through transmembrane pores: The roles of cholesterol and anionic lipids. Soft Matter.

[80] Oh K.J., Klein M.L. (2009). Effects of halothane on dimyristoylphosphatidylcholine lipid bilayer structure: A molecular dynamics simulation study. Bull. Korean Chem. Soc..

[81] Klauda J.B., Venable R.M., Freites J.A., O’Connor J.W., Tobias D.J., Mondragon-Ramirez C., Vorobyov I., MacKerell A.D., Pastor R.W. (2010). Update of the CHARMM all-atom additive force field for lipids: Validation on six lipid types. J. Phys. Chem. B.

[82] Pastor R.W., MacKerell A.D. (2011). Development of the CHARMM force field for lipids. J. Phys. Chem. Lett..

[83] Jorgensen W.L., Chandrasekhar J., Madura J.D., Impey R.W., Klein M.L. (1983). Comparison of simple potential functions for simulating liquid water. J. Chem. Phys..

[84] Kim S., Lee J., Jo S., Brooks C.L., Lee H.S., Im W. (2017). CHARMM-GUI ligand reader and modeler for CHARMM force field generation of small molecules. J. Comput. Chem..

[85] Humphrey W., Dalke A., Schulten K. (1996). VMD: Visual molecular dynamics. J. Mol. Graph..

[86] Jo S., Kim T., Iyer V.G., Im W. (2008). CHARMM-GUI: A web-based graphical user interface for CHARMM. J. Comput. Chem..

[87] Berendsen H.J.C., Postma J.P.M., van Gunsteren W.F., DiNola A., Haak J.R. (1984). Molecular dynamics with coupling to an external bath. J. Chem. Phys..

[88] Nosé S. (1984). A molecular dynamics method for simulations in the canonical ensemble. Mol. Phys..

[89] Hoover W.G. (1985). Canonical dynamics: Equilibrium phase-space distributions. Phys. Rev. A.

[90] Parrinello M., Rahman A. (1981). Polymorphic transitions in single crystals: A new molecular dynamics method. J. Appl. Phys..

[91] Hess B. (2008). P-LINCS: A parallel linear constraint solver for molecular simulation. J. Chem. Theory Comput..

[92] Miyamoto S., Kollman P.A. (1992). Settle: An analytical version of the SHAKE and RATTLE algorithm for rigid water models. J. Comput. Chem..

[93] Essmann U., Perera L., Berkowitz M.L., Darden T., Lee H., Pedersen L.G. (1995). A smooth particle mesh Ewald method. J. Chem. Phys..

[94] Dickson C.J., Rosso L., Betz R.M., Walker R.C., Gould I.R. (2012). GAFFlipid: A general amber force field for the accurate molecular dynamics simulation of phospholipid. Soft Matter.

[95] Dickson C.J., Madej B.D., Skjevik Å.A., Betz R.M., Teigen K., Gould I.R., Walker R.C. (2014). Lipid14: The amber lipid force field. J. Chem. Theory Comput..

[96] Nagle J.F. (1993). Area/lipid of bilayers from NMR. Biophys. J..

[97] Petrache H.I., Dodd S.W., Brown M.F. (2000). Area per lipid and acyl length distributions in fluid phosphatidylcholines determined by 2H NMR spectroscopy. Biophys. J..

[98] Miñones J., Rodríguez Patino J.M., Dynarowicz-Latka P., Carrera C. (2002). Structural and topographical characteristics of dipalmitoyl phosphatidic acid in Langmuir monolayers. J. Colloid Interface Sci..

[99] Stidder B., Fragneto G., Roser S.J. (2007). Structure and stability of DPPE planar bilayers. Soft Matter.

[100] Tiwary P., Parrinello M. (2015). A time-independent free energy estimator for metadynamics. J. Phys. Chem. B.

[101] Dama J.F., Parrinello M., Voth G.A. (2014). Well-tempered metadynamics converges asymptotically. Phys. Rev. Lett..

[102] Nencini R., Ollila O.H.S. (2022). Charged small molecule binding to membranes in MD simulations evaluated against NMR experiments. J. Phys. Chem. B.

[103] Krämer A., Ghysels A., Wang E., Venable R.M., Klauda J.B., Brooks B.R., Pastor R.W. (2020). Membrane permeability of small molecules from unbiased molecular dynamics simulations. J. Chem. Phys..

