# Supplementary material for: Influence of Membrane Composition on the Passive Membrane Penetration of Industrially Relevant NSO-Heterocycles
Source: Int J Mol Sci. 2025 Aug 1;26(15):7427. doi: 10.3390/ijms26157427 (PMC12347800; doi:10.3390/ijms26157427)

International Journal of
Molecular Sciences

MDPI

SUPPORTING INFORMATION

Zsófia Borbála Rózsa[1], Tamás Horváth[1,2], Béla Viskolcz[1,2], Milán Szőri[1*]

[1] Institute of Chemistry, University of Miskolc, Egyetemváros A/2, H-3515 Miskolc, Hungary; zsofia.borbala.rozsa@uni-miskolc.hu (ZBR); tamas.horvath@uni-miskolc.hu (TH); bela.viskolcz@uni-miskolc.hu (BV)

[2] Higher Education and Industrial Cooperation Centre, University of Miskolc, H-3515 Miskolc, Hungary; tamas.horvath@uni-miskolc.hu (TH); bela.viskolcz@uni-miskolc.hu (BV)

* Correspondence: milan.szori@uni-miskolc.hu (MS)

Figure S1 Change in box dimensions over the last 300 ns of simulation for each membrane system. Data were smoothed using a rolling average with a window size of 100 frames.

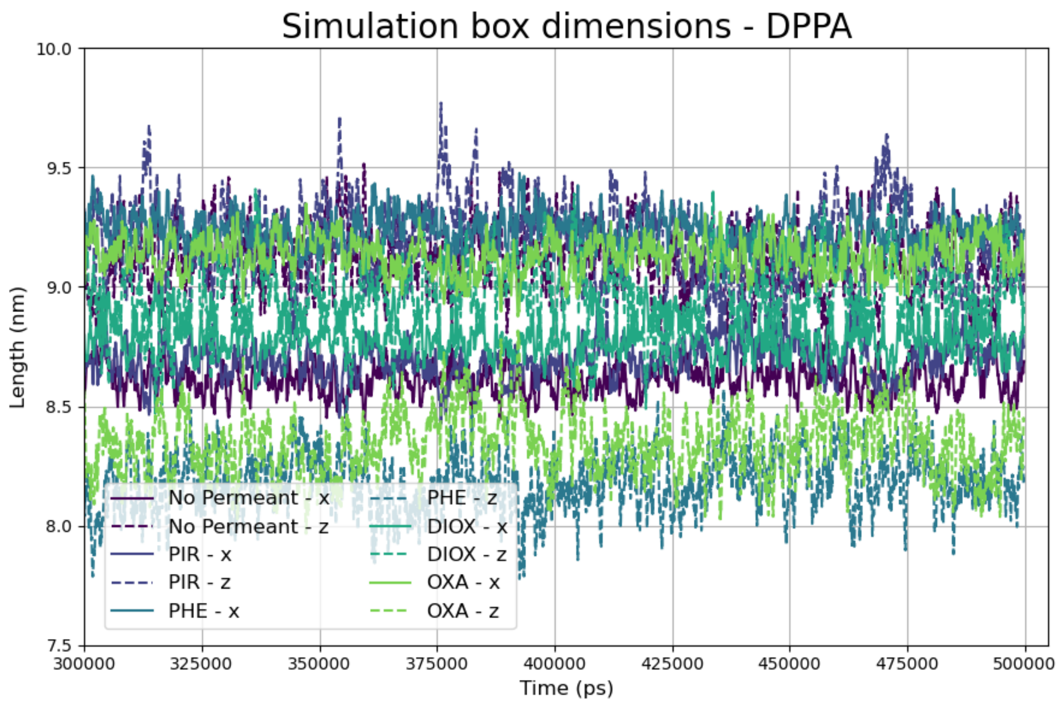

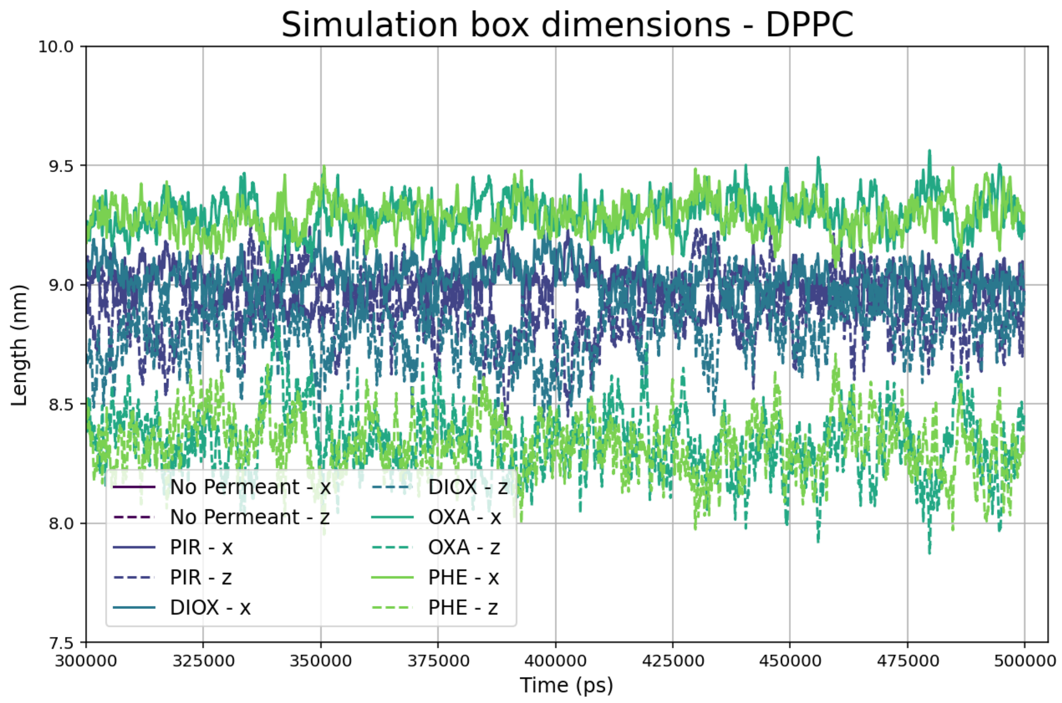

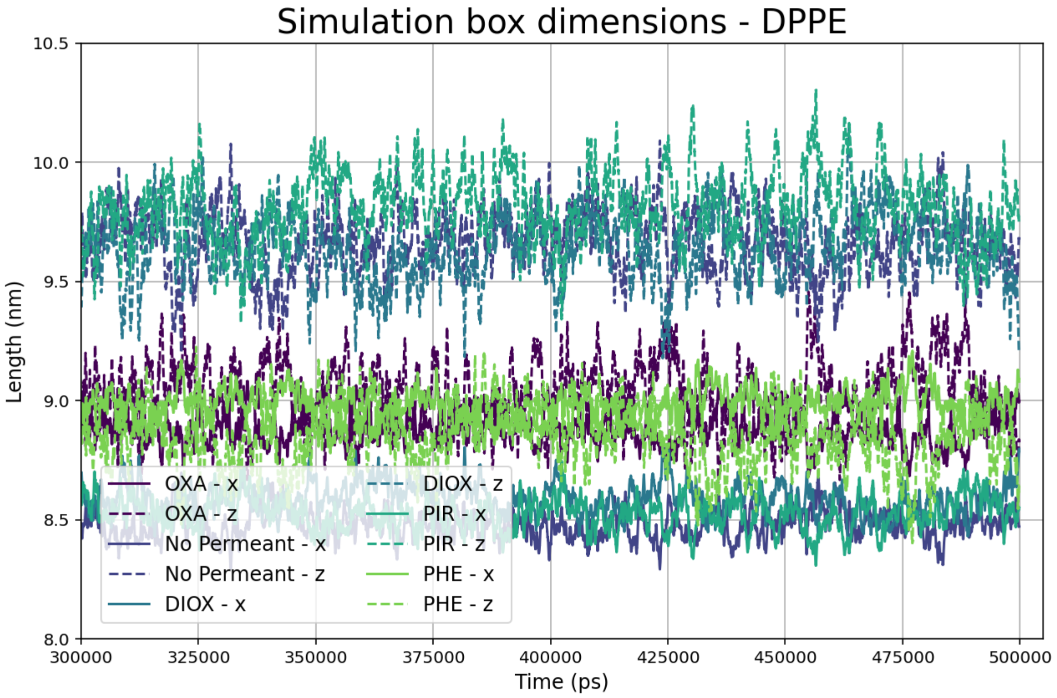
Simulation box dimensions - DPPE
Length (nm)
Time (ps)
OXA - x
OXA - z
No Permeant - x
No Permeant - z
DIOX - x
DIOX - z
PIR - x
PIR - z
PHE - x
PHE - z
10.5
10.0
9.5
9.0
8.5
8.0
300000
325000
350000
375000
400000
425000
450000
475000
500000

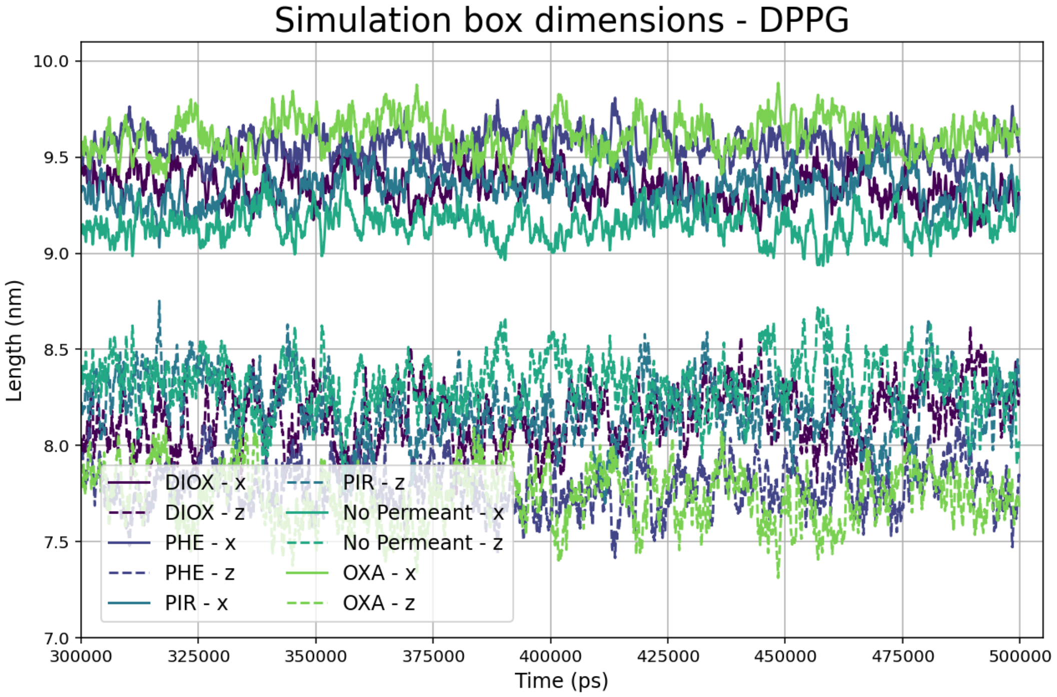
Simulation box dimensions - DPPG
Length (nm)
Time (ps)
DIOX - x
DIOX - z
PHE - x
PHE - z
PIR - x
PIR - z
No Permeant - x
No Permeant - z
OXA - x
OXA - z
10.0
9.5
9.0
8.5
8.0
7.5
7.0
300000
325000
350000
375000
400000
425000
450000
475000
500000

Figure S2 Mass Density Profile of the investigated membrane-NSO-HET systems.

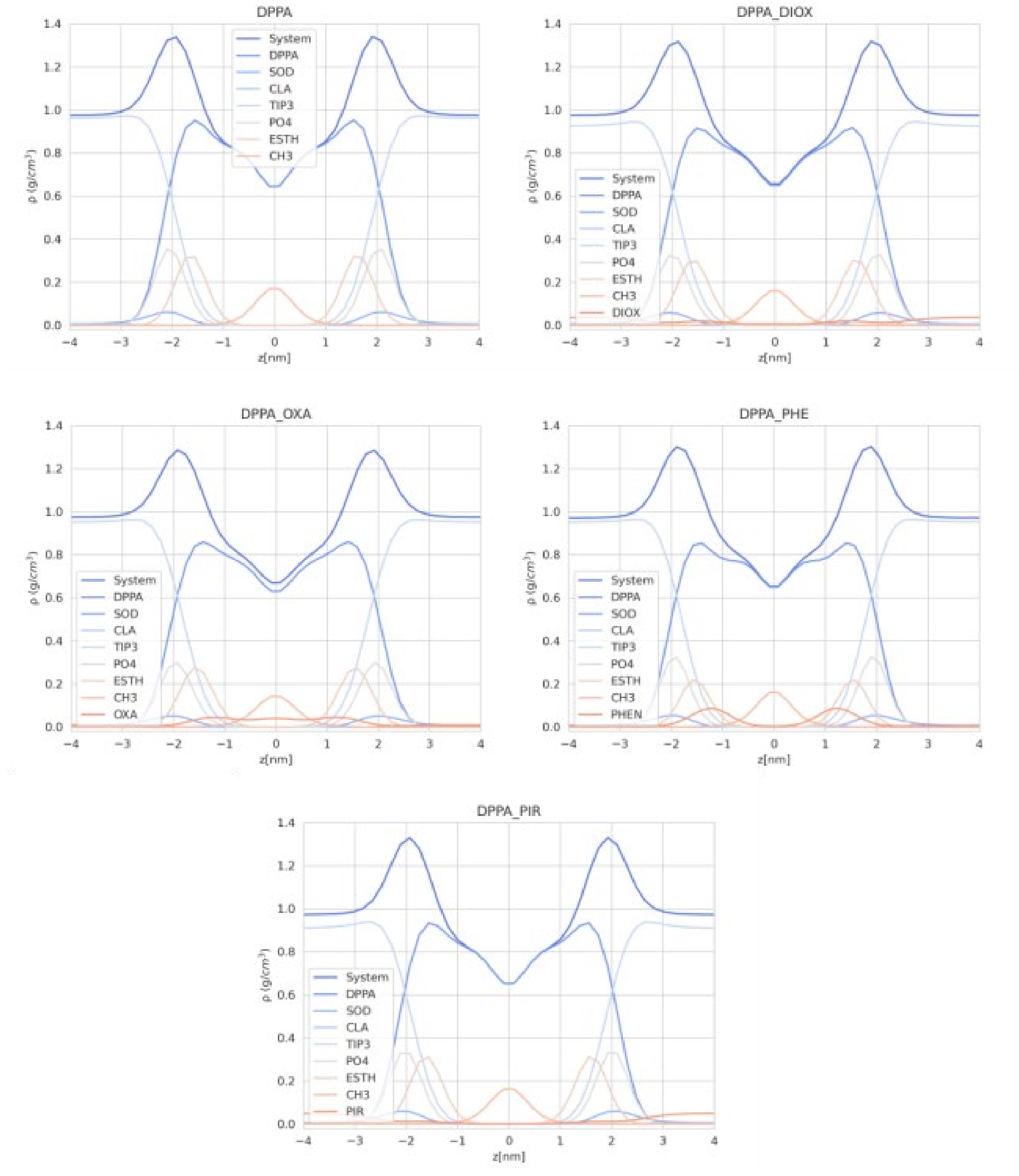

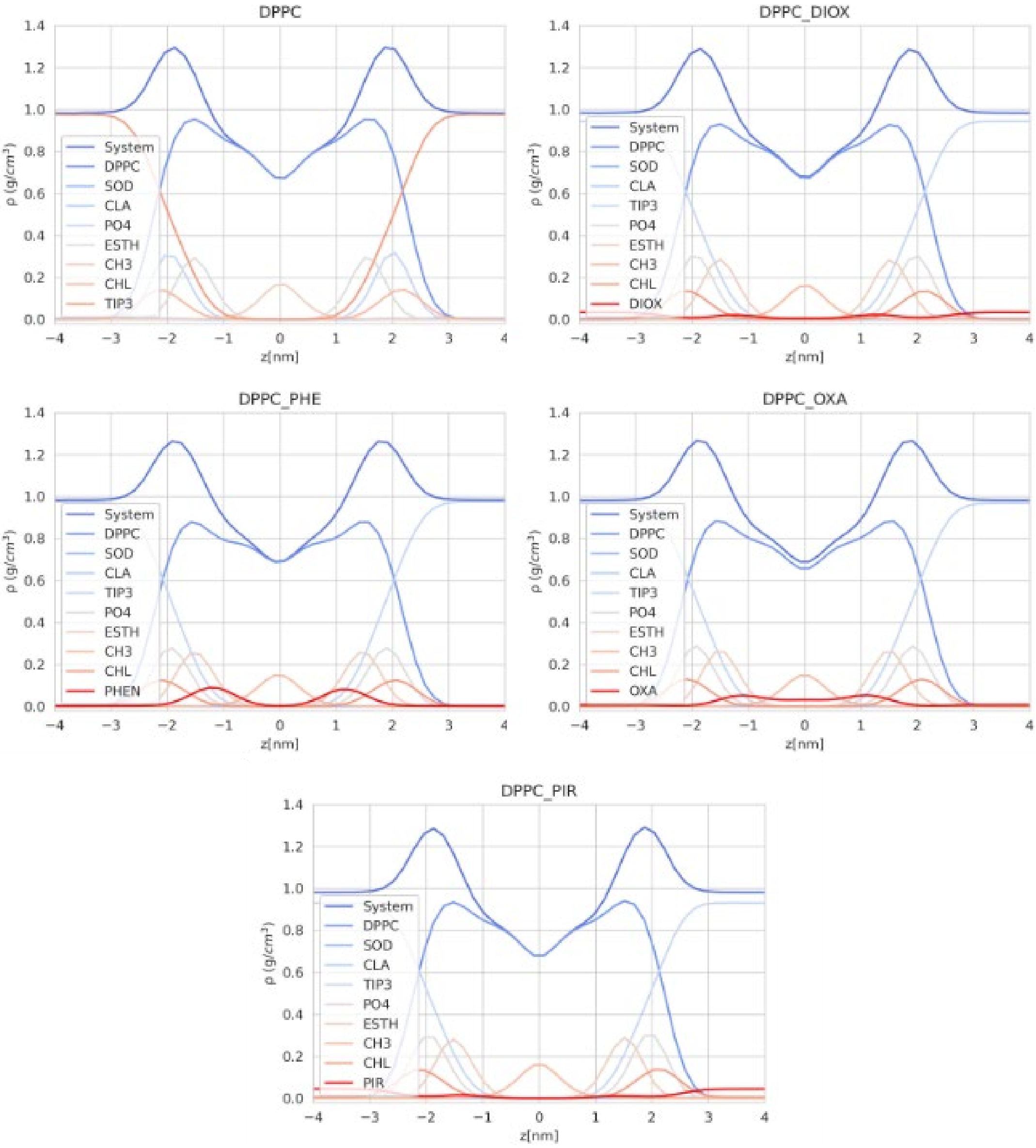
DPPC
DPPC_DIOX
DPPC_PHE
DPPC_OXA
DPPC_PIR
ρ (g/cm³)
z[nm]
System
DPPC
SOD
CLA
TIP3
PO4
ESTH
CH3
CHL
DIOX
PHEN
OXA
PIR

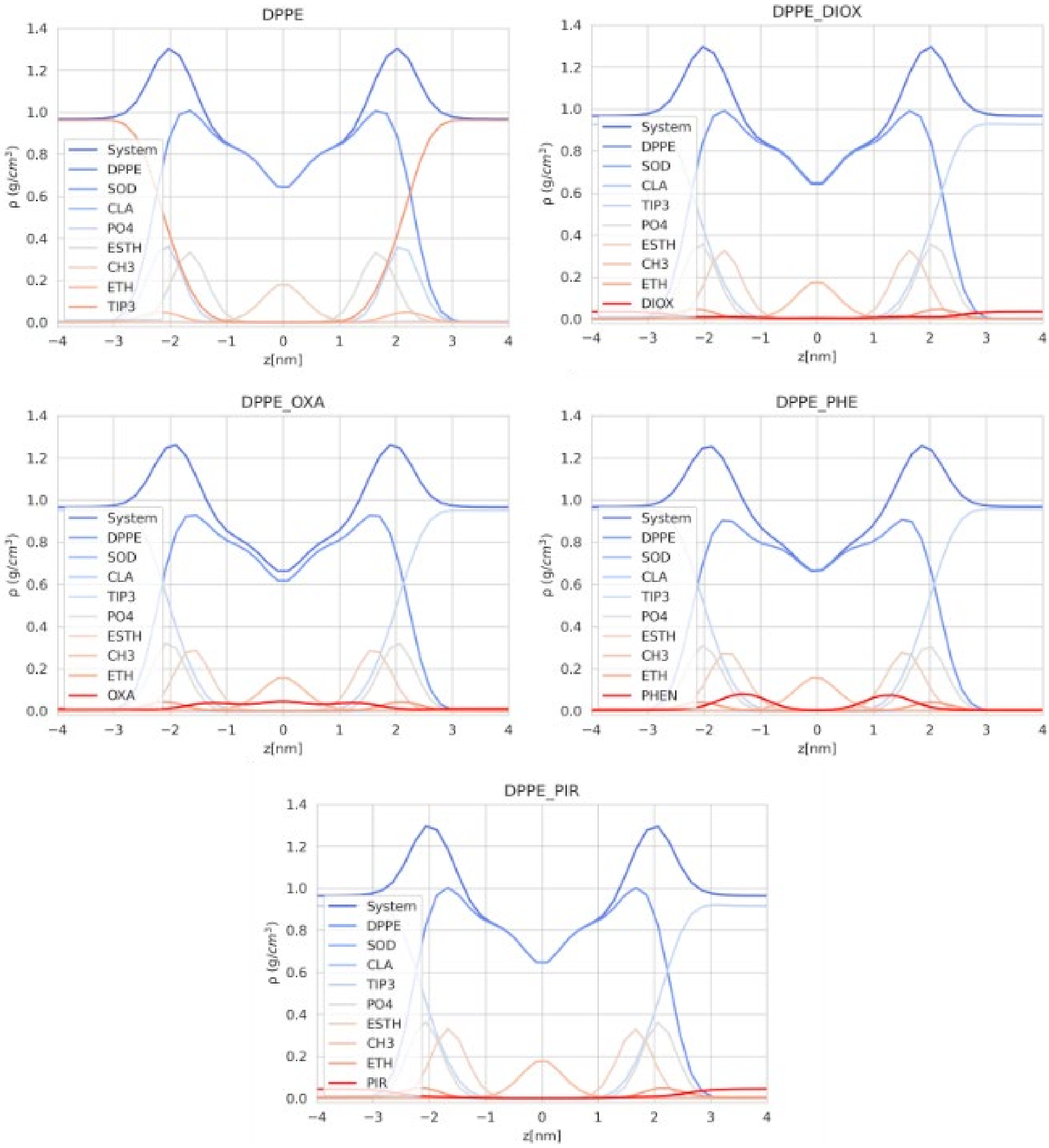
DPPE
System
DPPE
SOD
CLA
PO4
ESTH
CH3
ETH
TIP3
DPPE_DIOX
System
DPPE
SOD
CLA
TIP3
PO4
ESTH
CH3
ETH
DIOX
DPPE_OXA
System
DPPE
SOD
CLA
TIP3
PO4
ESTH
CH3
ETH
OXA
DPPE_PHE
System
DPPE
SOD
CLA
TIP3
PO4
ESTH
CH3
ETH
PHEN
DPPE_PIR
System
DPPE
SOD
CLA
TIP3
PO4
ESTH
CH3
ETH
PIR
ρ (g/cm³)
z[nm]

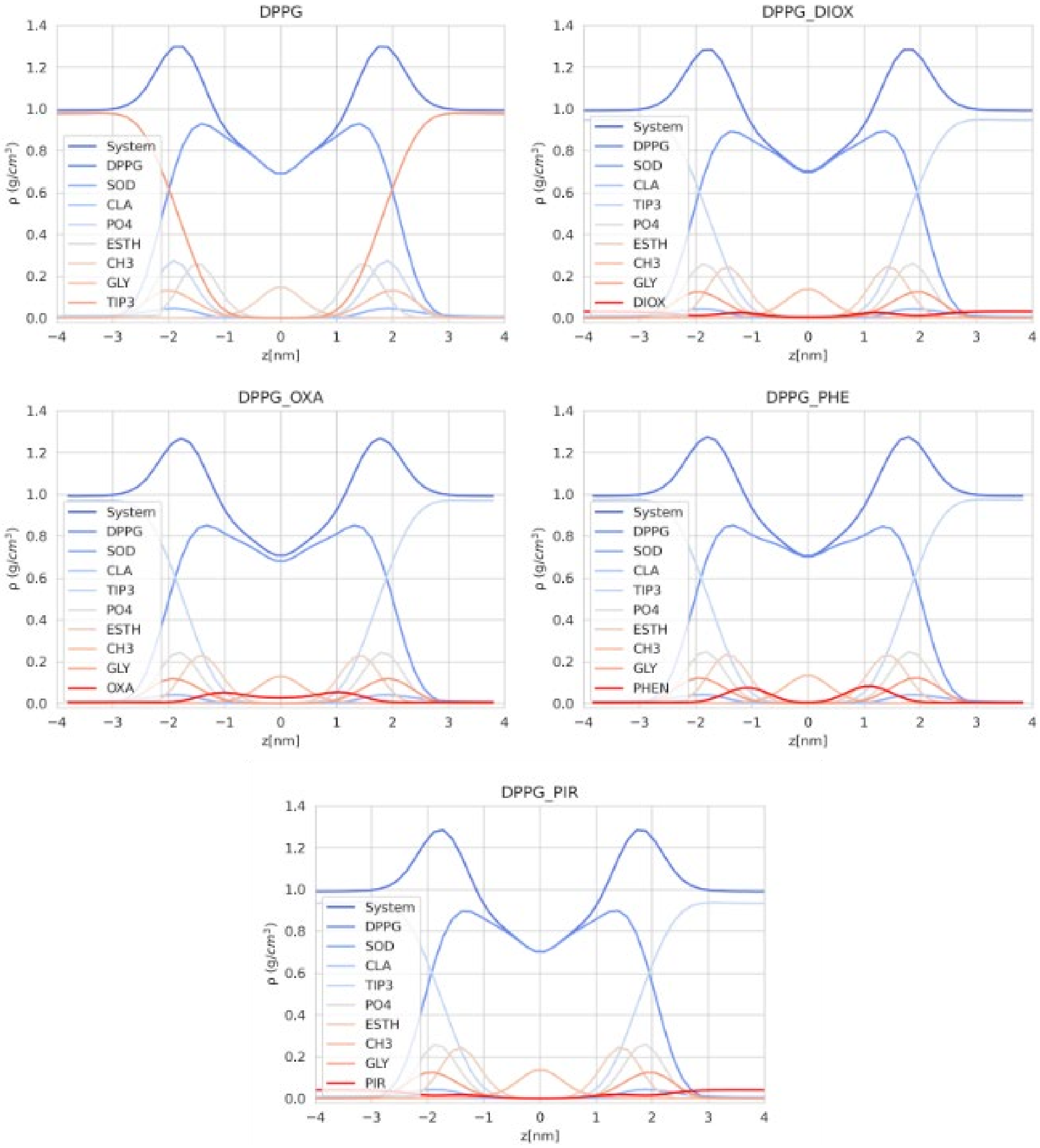

Figure S3 Comparison of membrane penetration free energy profiles obtained using the density-based method and the adaptive biasing approach (AWH) for all investigated compounds. The good agreement between the two methods supports the reliability and consistency of the PMF results used in this study.

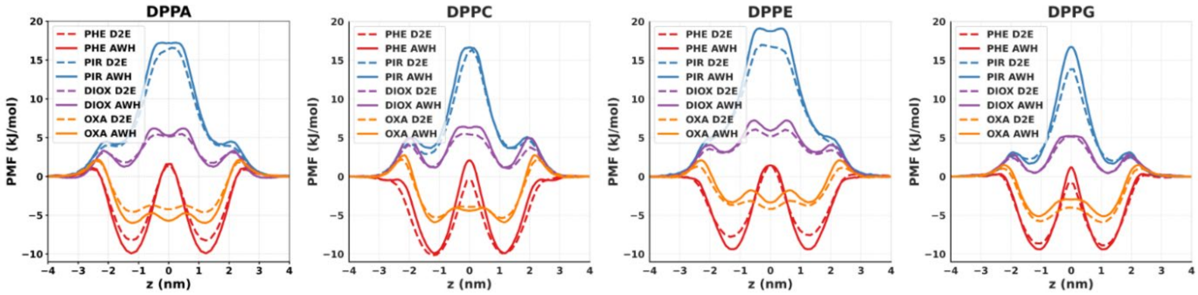

Supplement: Supplementary file 1 [file ijms-26-07427-s001.zip › ijms-3765829-supplementary.pdf]
